# Aortic Annular Enlargement during Aortic Valve Replacement

**DOI:** 10.3889/oamjms.2016.098

**Published:** 2016-09-02

**Authors:** Selman Dumani, Ermal Likaj, Laureta Dibra, Stavri Llazo, Ali Refatllari

**Affiliations:** *University Hospital Center “Mother Theresa”, Tirana, Albania*

**Keywords:** AAE-aortic annulus enlargement, Manouguian technique, PPM-patient - prosthesis mismatch, AVR-aortic valve replacement, iEOA-indexed effective orifice area

## Abstract

In the surgery of aortic valve replacement is always attempted, as much as possible, to implant the larger prosthesis with the mains goals to enhance the potential benefits, to minimise transvalvular gradient, decrease left ventricular size and avoid the phenomenon of patient-prosthesis mismatch. Implantation of an ideal prosthesis often it is not possible, due to a small aortic annulus. A variety of aortic annulus enlargement techniques is reported to avoid patient-prosthesis mismatch. We present the case that has submitted four three times open heart surgery. We used Manouguian technique to enlarge aortic anulus with excellent results during the fourth time of surgery.

## Introduction

In the surgery of aortic valve replacement is always attempted, as much as possible, to implant the larger prosthesis with the mains goals to enhance the potential benefits, to minimise transvalvular gradient, decrease left ventricular size and avoid the phenomenon of patient-prosthesis mismatch. Implantation of an ideal prosthesis often it is not possible, due to a small aortic annulus. A variety of aortic annulus enlargement techniques is reported to avoid patient-prosthesis mismatch. We present the case that has submitted four three times open heart surgery. We used Manouguian technique to enlarge aortic annulus with excellent results during the fourth time of surgery.

## Case Presentation

Patient Z.M. 52 years old was admitted to our service with the diagnosis: Status post replacement of mitral and aortic valve, dysfunction of the aortic prosthetic valve, heart failure NYHA III-IV.

The patient was operated three times: 1990- open mitral commissurotomy for rheumatic valvular disease, 1997 mitral and aortic valve replacement with a mechanical prosthesis, 2004 replacement of aortic prosthesis for prosthetic dysfunction.

The patient at admission had the prosthesis Sorin Nr.19 in the aortal position. The echocardiographic data present normal function and diameters of left ventricle, while mean aortic trans-prosthetic gradient was 70 mmHg. The mobility of prosthetic leaflets was normal. There were no data for prosthetic panus or thrombus. There was a normal function of the mitral prosthesis. The patient was in NYHA III clinical status. It was clear that the main problem was the patient-prosthesis mismatch. The patient was in severe patient – prosthesis mismatch. The calculated indexed effective orifice area was 0.64 cm^2/m^2.

In these circumstances, it was established the indication for redo surgery to resolve the problem of patient - prosthesis mismatch. The patient underwent routine preoperative examinations to be prepared for intervention.

Intervention was performed through median sternotomy with standard cardiopulmonary bypass and systemic hypothermia to 32°C. An oblique aortotomy was performed and myocardial protection was provided by intermittent antegrade cristaloidcardioplegia delivered directly into the coronary ostia. We have inspected carefully the prosthesis and there was no panus or thrombus near the prosthesis. In these conditions, we decided that the best solution was the replacement of the prosthesis with a new one and in the same time doing enlargement of the aortic annulus. After removing the old prosthesis, aortic annulus enlargement was done using Manouguian technique [1]. Aortotomy was extended through annulus into the fibrous trigone between the noncoronary cusp and the left coronary cusp to the subaortic curtain and anterior mitral valve leaflet. This defect was closed using the synthetic Teflon patch.

**Figure 1:**
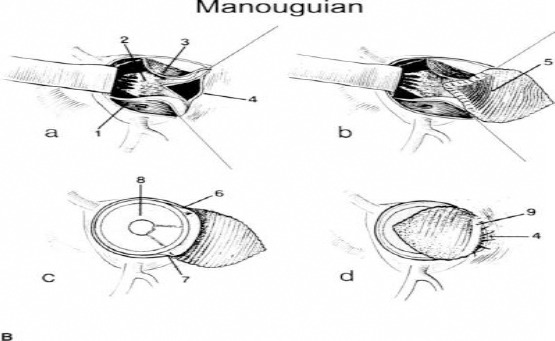
Schematic Manouguian Technique

We implanted SJM prosthesis Nr 21. Aortic cross-clamping time was 110 minutes. Cardiopulmonary bypass time was 130 minutes. The patient did the usual postoperative course as standard aortic valve replacement.

**Figure 2:**
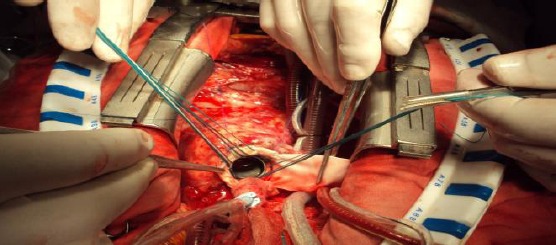
Photo during intervention

## Discussion

Rahimtola et al [2] presented for the first time since 1978 the issue of prosthesis - patient mismatch which is defined as a condition in which the effective surface of the prosthesis is less than that of the normal patient valve.

The most accurate and used a parameter to define patient - prosthesis mismatch (PPM) actually is the indexed prosthetic effective orifice area (iEOA) that is the ratio of the orifice area of the prosthesis (EOA) with the patient’s body surface area (BSA). Based on these values EOAi ≤ 0.85 cm^2/m^2 is regarded as the threshold for the occurrence of PPM to continue with moderate PPM when iEOA value is between 0.65 cm^2/m^2-0.85 cm^2/m^2 and severe when iEOA < 0.65 cm^2/m^2. The patient-prosthesis mismatch is common phenomenon during aortic valve replacement. The reported incidence varies 2-11 % [3].

There are four ways to resolve the problem of mismatch: implantation of the stentless prosthesis, homograft, autograft and aortic annulus enlargement (AAE) [1, 4-6].

The first three are associated with an increased operative mortality and morbidity [3]. Aortic annulus enlargement remains the more simple and reproducible surgical procedure to avoid this phenomenon.

Aortic annulus enlargement is an additional surgical procedure and it is performed in an anatomical area with high risk of bleeding. These facts have provoked the debate about the impact of this procedure on the early results of aortic valve surgery.

Aortic annulus enlargement procedure [7, 8] does not affect negatively early results of aortic valve surgery in terms of hospital mortality and morbidity even while, due to the complexity of the procedure, cross-clamping and cardiopulmonary bypass times are relatively longer than in standard aortic valve replacement. In this context Coutinho et al [7] recommend strongly the necessity to involve the aortic annulus enlargement procedure as part of operating strategy whenever is necessary during aortic valve replacement in patients with small aortic annulus. These suggestions are supported by other authors with a smaller contingent of patients operated that have realised aortic annulus enlargement [9, 10].

There are authors that analysing their results report higher mortality and morbidity in the group with AAE. They criticise the routine use of the aortic annulus enlargement and recommend being careful in the management of patient –prosthesis mismatch [11].

Mayo Clinic presented one of the largest studies where are involved 2366 patients in which 10.5 % of patients have been the subject of aortic annulus enlargement during aortic valve replacement. This study shows that the small number of a prosthesis implanted is an independent important risk factor in the early operative results while the aortic annulus enlargement procedure does not influence perioperative mortality and morbidity [12].

The accurate indications for AAE in the contingent of patients who are at risk to show to have postoperative patient-prosthesis mismatch improved significantly results of aortic valve surgery. Peterson et al report that in the modern era AAE is importantly improved. They present a comparison between two large groups of patients operated different periods find out a significant decline in hospital mortality from 7.5 to 3 % respectively for the periods 1995-2000 and 2001-2005 [13]. Aortic annulus enlargement, in a multifactorial analysis, is not a risk factor in aortic valve surgery and is recommended to be performed specifically in separate contingents of patients as in young patients and in those with reduced function of the left ventricle.

In our case, the early and late results of aortic annulus enlargement demonstrate effective solution of patient - prosthesis mismatch. The iEOA is 0.84 cm^2/m^2. The aortic cross-clamping and cardiopulmonary bypass time were longer in comparison with standard aortic valve replacement but the patient did very good postoperative period. We had no excessive bleeding and usual respiratory assistance, intensive care unit and postoperative hospital stay in comparison with standard aortic valve replacement. The early postoperative period was very good. The patient is in very good health after hospital discharge. She is in NYHA class I and does normal life for her age.

Manouguian technique is used successfully in a significant number of patients operated in our service, but this is not the topic of this presentation. This fact encourages us to involve the aortic annulus enlargement procedure in patient with high risk of patient - prosthesis mismatch, during aortic valve replacement.

In conclusion, aortic annulus enlargement during aortic valve replacement according to Manouguian is a safe technique that solves the problem of patient- prosthesis mismatch.

## References

[1] Manouguian S, Seybold-Epting W (1979). Patch enlargement of the aortic valve ring by extending the aortic incision to the anterior mitral leaflet. J Thorac Cardiovasc Surg.

[2] Rahimtoola SH (1978). The problem of valve prosthesis—patient mismatch. Circulation.

[3] Pibarot P, Dumesnil JG (2006). Prosthesis-patient mismatch: definition, clinical impact, and prevention. Heart.

[4] Nu-ez L, Gil Aguado M, Pinto GA, Larrea JL (1983). Enlargement of the Aortic Annulus by Resecting the Commissure Between the Left and Noncoronary Cusps. Tex Heart Inst J.

[5] Nicks R, Cartmill T, Bernstein L (1970). Hypoplasia of the aortic root: the problem of aortic valve replacement. Thorax.

[6] Kinsley RH, Antunes MJ (1983). Enlargement of the narrow aortic root and oblique insertion of a St. Jude prosthesis. Br Heart J.

[7] Coutinho GF, Correia PM, Paupe´rio G, De Oliveira F, Antunes MJ (2011). Aortic root enlargement does not increase the surgical risk and short-term patient outcome?. European Journal of Cardio-thoracic Surgery.

[8] Castro LJ, Arcidi MJ, Fisher AL, Gaudiani VA (2002). Routine enlargement of the small aortic root: a preventive strategy to minimize mismatch. Ann Thorac Surg.

[9] St Rammos K, Ketikoglou DG, Koullias GJ, Tsomkopoulos SG, Rammos CK, Argyrakis NP (2006). The Nicks–Nunez posterior enlargement in the small aortic annulus: immediate–intermediate results. Interactive CardioVascular and Thoracic Surgery.

[10] Srivastava DK, Sanki P, Bhattacharya S, Siddique JV (2014). Strategy to avoid patient-prosthesis mismatch: aortic root enlargement. Asian Cardiovasc Thorac Ann.

[11] LaPar DJ, Ailawadi G, Bhamidipati CM, Stukenborg G, Crosby IK, Kern JA, Kron IL (2011). Small Prosthesis Size in Aortic Valve Replacement Does Not Affect Mortality. Ann Thorac Surg.

[12] Dhareshwar J, Sundt TM, Dearani JA, Schaff HV, Cook DJ, Orszulak TA (2007). Aortic root enlargement: what are the operative risks?. J Thorac Cardiovasc Surg.

[13] Peterson MD, Borger MA, Feindel CM, David TE (2007). Aortic Annular Enlargement During Aortic Valve Replacement: Improving Results with Time. Ann Thorac Surg.

